# Passive Film Properties of Bimodal Grain Size AA7075 Aluminium Alloy Prepared by Spark Plasma Sintering

**DOI:** 10.3390/ma13143236

**Published:** 2020-07-21

**Authors:** Wenming Tian, Zhonglei Li, HuiFeng Kang, Fasong Cheng, Fangfang Chen, Guoxing Pang

**Affiliations:** 1 School of Materials Engineering, North China Institute of Aerospace Engineering, No.133 Aimindong Road, Langfang 065000, China; lzl1998@nciae.edu.cn (Z.L.); huifkang@163.com (H.K.); Chenfangfang619@163.com (F.C.); pangguoxing@nciae.edu.cn (G.P.); 2 Heibei Key Laboratory of Trans-Media Aerial Underwater Vehicle, North China Institute of Aerospace Engineering, No.133 Aimindong Road, Langfang 065000, China; 3 Aero Engine Corporation of China (AECC) Guizhou Liyang Aviation Power CO., LTD., Guiyang 550014, China; m18720062093_2@163.com

**Keywords:** aluminium alloy, passive film, corrosion, electrochemical, powder metallurgy

## Abstract

The bimodal-grain-size 7075 aluminium alloys containing varied ratios of large and small 7075 aluminium powders were prepared by spark plasma sintering (SPS). The large powder was 100 ± 15 μm in diameter and the small one was 10 ± 5 μm in diameter. The 7075 aluminium alloys was completely densified under the 500 °C sintering temperature and 60 MPa pressure. The large powders constituted coarse grain zone, and the small powders constituted fine grain zone in sintered 7075 aluminium alloys. The microstructural and microchemical difference between the large and small powders was remained in coarse and fine grain zones in bulk alloys after SPS sintering, which allowed for us to investigate the effects of microstructure and microchemistry on passive properties of oxide film formed on sintered alloys. The average diameter of intermetallic phases was 201.3 nm in coarse grain zone, while its vale was 79.8 nm in fine grain zone. The alloying element content in intermetallic phases in coarse grain zone was 33% to 48% higher than that on fine grain zone. The alloying element depletion zone surrounding intermetallic phases in coarse grain zone showed a bigger width and a more severe element depletion. The coarse grain zone in alloys showed a bigger electrochemical heterogeneity as compared to fine grain zone. The passive film formed on coarse grain zone had a thicker thickness and a point defect density of 2.4 × 10^24^ m^−3^, and the film on fine grain zone had a thinner thickness and a point defect density of 4.0 × 10^23^ m^−3^. The film resistance was 3.25 × 10^5^ Ωcm^2^ on coarse grain zone, while it was 6.46 × 10^5^ Ωcm^2^ on fine grain zone. The passive potential range of sintered alloys increased from 457 mV to 678 mV, while the corrosion current density decreased from 8.59 × 10^−7^ A/cm^2^ to 6.78 × 10^−7^ A/cm^2^ as fine grain zone increasing from 0% to 100%, which implied that the corrosion resistance of alloys increased with the increasing content of fine grains. The passive film on coarse grain zone exhibited bigger corrosion cavities after pitting initiation compared to that on fine grain zone. The passive film formed on fine grain zone showed a better corrosion resistance. The protectiveness of passive film was mainly determined by defect density rather than the thickness in this work.

## 1. Introduction

Aluminium and its alloys are commonly used in automobile and aerospace industries, because of their attractive properties, such as high strength and good corrosion resistance [1,2,3]. Some alloying elements, including Li, Si, Mg, Cu, and Zn, are introduced in aluminium to primarily improve its mechanical properties [4,5]. Aluminium metallic composites that are mainly reinforced by SiC, Al_2_O_3_, AlN, Si_3_N_4_, and BN particles are also increasingly used in industries [6,7]. However, because the traditional fabrication approaches of aluminium alloys and composites often encounter the segregation and coarsening of microstructures, new kinds of technologies, such as spark plasma sintering (SPS), are employed to manufacture aluminium alloys. SPS is suitable for preparing high quality metals with very fine microstructures, due to its lower sintering temperature, faster heating rate, and shorter cycle time than other methods [5,6,7,8,9]. Its heating arises from Joule heat, which is caused by high current passing through the sites having large ohm resistance. Therefore, the Joule heat concentrates at gaps and contact points between powders, which results in a high temperature far exceeding the setting value. The powder surface is melted and the core remains solid and cool [5]. Besides, the high pulsed current generates localized micro-plasma in the gaps between powders, which can clean the powder surface and increase atom diffusion. Thus, the SPS can reduce pores and prepares completely densified metals [10,11]. Ameyama et al. used such s technique to manufacture the “Harmonic Structure” (also named as bimodal-grain-size) metals, which showed good strength and ductility [12,13,14]. The 2024 alloy with ultrafine lamellar structure was prepared by mechanical milling and SPS, and exhibited remarkably improved strength [5]. The SPS sintered metals definitely shows improved mechanical properties as compared to cast metals, even though the metallic powders do not undergo mechanical milling [15,16,17].

The cooling rate of powders is in direct proportion to their specific surface. Therefore, small metallic powders have a faster cooling rate during solidification from molten alloy droplet, since they have a bigger specific surface than the large one. Different sized metallic powders usually exhibit different microstructures, such as grain size and second phases distribution. The SPS sintered alloys maintain the microstructural difference of powders after sintering, because of the low sintering temperature and fast heating rate [10,11]. The size of metallic powders can change the microstructures of sintered aluminium alloys and, thus, affects their mechanical and corrosion performance [18,19].

In this study, the bimodal-grain-size AA7075 aluminium alloys with varied ratios of powders of different sizes, large (*Φ*100 μm) and small (*Φ*10 μm), were prepared by SPS. The effects of powder size on passive film properties of sintered alloys were revealed. The microstructural and microchemical difference between large and small powders were maintained in sintered aluminium alloys. Large powders constituted coarse grain zone, while small powders constituted fine grain zone in sintered alloys. The microstructure and passive properties of such alloys were investigated by physical and electrochemical measurements. The relevance between the passive properties and microstructure of alloys was discussed, and would provide guidance to designing new kinks of aluminium alloys with high corrosion resistance and good mechanical properties. This study also introduced a new method for preparing bimodal-grain-size aluminium alloys.

## 2. Experimental

### 2.1. Materials and Processing

Six kinds of bulk alloys, named as AL1 to AL6, were sintered with two sizes of 7075 aluminium powders. AL1 contained 100 wt% large powder, AL2 contained 80 wt% large powder and 20 wt% small powder, AL3 contained 60 wt% large powder and 40 wt% small powder, AL4 contained 40 wt% large powder and 60 wt% small powder, AL5 contained 20 wt% large powder and 80 wt% small powder, and AL6 contained 100 wt% small powder Both the large and small powders were prepared by rotating disk atomization and provided by Haotian company, Shanghai China. The diameter of powders was determined by rotational speed of disk, and a faster speed resulted in a smaller powder diameter. The diameters of large and small powders were 100 ± 15 μm and 10 ± 5 μm, respectively, which were measured by Nanomeasure software. In the sintered alloys, the small powders assembled the fine grain zone, and the large powders assembled the coarse grain zone. Prior to sintering, the large and small powders were wet mixed in an acetone ultrasonic bath for 2 h according to the weight ratios mentioned above, and then vacuum dried at 50 °C for 12 h. The mixed aluminium powders were loaded in a cylindrical graphite die (with 15 mm inner diameter). Additionally, the SPS sintering was performed at 500 °C in vacuum with a dwell time of 1 min. and a heating rate of 50 °C/min. After SPS sintering, the bulk alloys were naturally cooled in vacuum. An axial pressure of 60 MPa was loaded during heating and cooling. All the alloy samples, from AL1 to AL6, were prepared at the same sintering condition. 

Each alloy sample was cut into 4 mm diameter cylinders and inlaid in phenolic resin to act as working electrode. Each electrode was spot welded with a copper wire on its back to provide electrical contact, and its non-working surface was sealed by epoxy resin. The working surface of electrode was grounded with 600 to 6000 # emery paper in turn, and then polished by 250 nm diamond spray suspension, and finally cleaned while using alcohol and deionized water. Before electrochemical tests, all of the electrodes were exposed to atmospheric air at 30 °C for 30 days to form a stable air-formed oxide film.

### 2.2. Microstructure Measurement

The metallographic morphologies of alloys were etched by Keller solution and then observed by KH-7700 optical microscope (HIROX company, Osaka, Japan). The microstructure of unsintered powders and sintered alloys were observed by Apollo 300 field-emission scanning electron microscope (SEM) (CamScan company, Cambridgeshire, UK) operating at 20 kV. The transmission electron microscope equipped with X-Max energy dispersive X-ray spectroscopy (EDS) (OXFORD company, Oxfordshire, UK) was employed to reveal detailed morphologies and composition of intermetallic phases in sintered alloys. The high angle annular dark field images taken by JEOL-2100F transmission electron microscope (TEM) (JEOL company, Tokyo, Japan) at 200 kV accelerating voltage were analyzed by ImageJ software 1.49t (National Institutes of Health, Bethesda, MD, USA) to obtain average diameter of intermetallic phases. Thin foils of bulk alloys that were prepared by ion beam thinning were used to TEM observation. The Dimension Icom Atomic Force Microscope (AFM) that was provided by Bruker company (Billica, MA, USA) was used to reveal the morphologies after passive film broken down with a contact model. The depth profiling of passive film was determined by AXIS Supra X-ray photoelectron spectroscopy (XPS) (Kratos company, Manchester, UK) that was equipped with an Ar^+^ ion gun. The diameter of X-ray spot was 500 μm.

### 2.3. Electrochemical Measurement

The electrochemical tests were performed on a CorrTest CS2350H electrochemical workstation (CorrTest company, Wuhan, China) with a tree-electrode system. A platinum sheet worked as the counter electrode and a saturated calomel electrode (SCE) worked as the reference electrode. The test solution was borate-buffered solution (pH = 7.2) containing 0.2 M Na_2_SO_4_ and 0.01 M NaCl. All of the potentials quoted in this work referred to the SCE.

Potentiodynamic polarization tests initiated at 200 mV below the open circuit potential (OCP) and scanned to the positive direction with a scan rate of 1 mV/s until the anodic current density reached 0.1 mA/cm^2^. The electrochemical impedance spectroscopy (EIS) was performed at the OCP while using a 10 mV AC stimulus signal in the frequency range from 10 mHz to 100 kHz. The Zsimpwin software 3.3d (AMETEK company, San Diego, CA, USA) was used to fit EIS data. The potential range of Mott–Schottky tests initiated at OCP and changed the potential to the positive direction with a scan rate of 20 mV/s until the potential reached 0 V. All of the electrochemical tests were performed after 6 h immersion when the alloys air-formed oxide film thoroughly hydrated, in order that the electrode obtains a steady state.

## 3. Results and Discussion

The bimodal-grain-size AA 7075 aluminium alloys containing varied ratios of large and small powders were prepared by SPS sintering. The passive film properties of such alloys were tested and discussed with the corresponding microstructures. This section reveals the microstructure and microchemistry of alloys as well as the electrochemical performance of passive films.

### 3.1. Microstructure of Alloys

**Figure 1:**
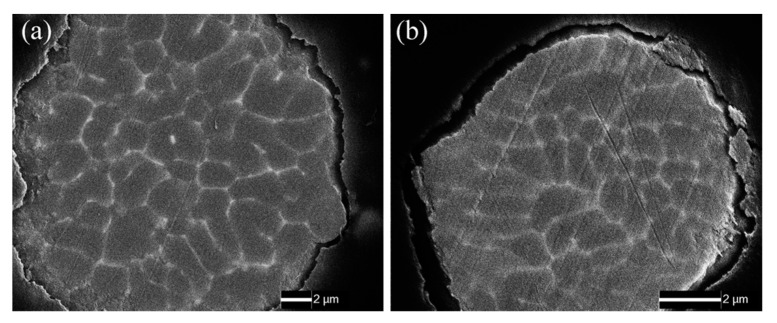
Scanning electron microscope (SEM) images of cross-section to unsintered 7075 aluminium powders, (**a**) large powder with 100 ± 15 μm diameter and (**b**) small powder with 10 ± 5 μm diameter.

Figure 1 shows the cross section of unsintered 7075 aluminium powders. Both the large (*Φ*100 μm) and small (*Φ*10 μm) powders were polycrystalline, because the brighter lines, which consist of heavy elements, such as Cu, Zn, Fe, and Cr, almost distributed along grain boundaries. The grains in large powder were several microns in diameter, while those in small powder had a diameter with hundreds of nanometers. Besides, the intermetallic phases (bright lines) in small powders also showed a narrower width than that in large powders. The molten alloy droplet cooling rate is in direct proportion to its specific surface area [20,21]. While, the small powder had 10 times bigger specific surface area as compared to the large one and, thus, a faster cooling rate resulted in refined structures (fine grains and intermetallic phases) in small powders.

**Figure 2:**
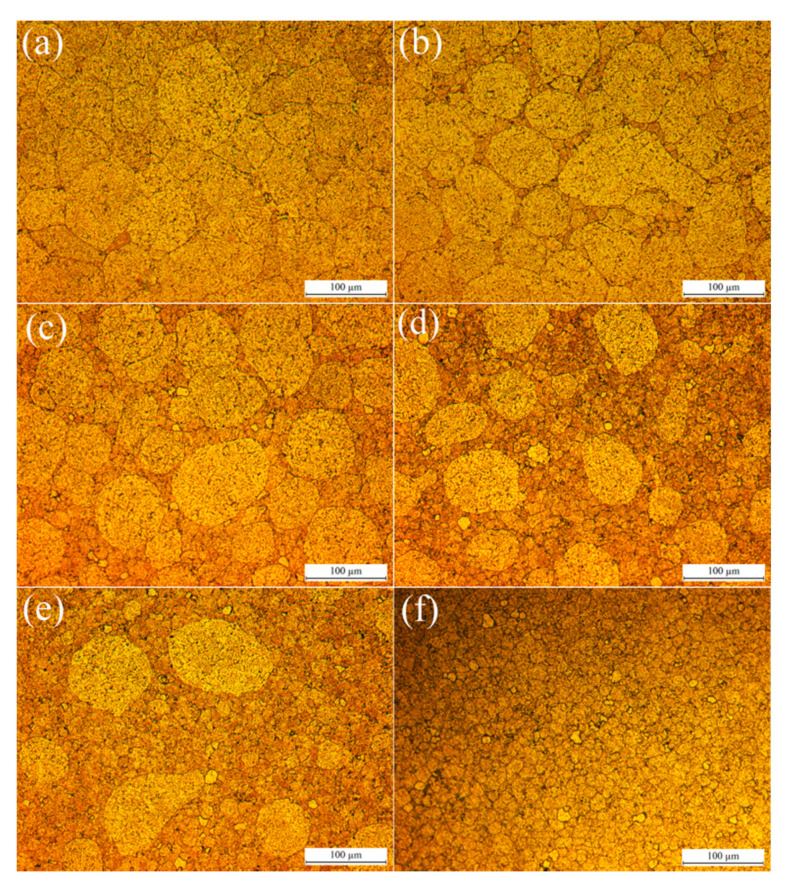
Typical metallographic images of sintered 7075 alloys, (**a**) AL1, (**b**) AL2, (**c**) AL3, (**d**) AL4, (**e**) AL5, and (**f**) AL6.

**Figure 3:**
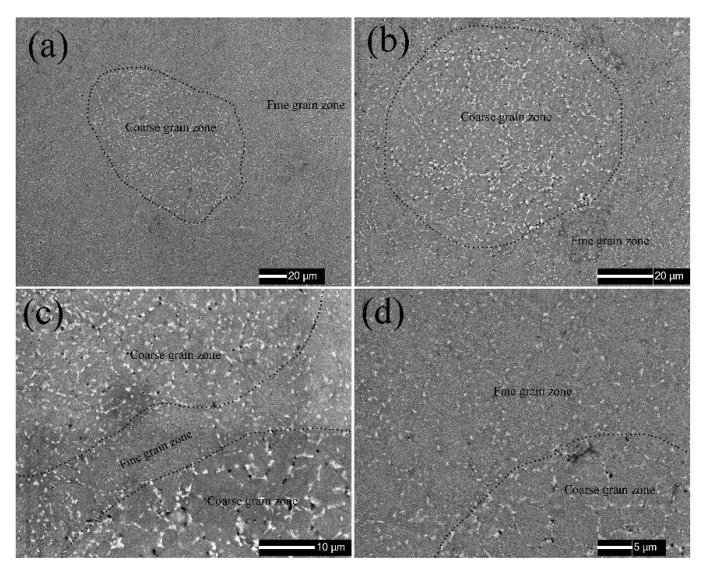
Typical SEM images of sintered 7075 alloys, (**a**,**b**) AL4, (**c**) AL3, and (**d**) AL5.

Figure 2 shows the typical metallographic features of sintered alloys, in where, both the large and small powders are dispersed very well in matrix, and no gaps as well as cracks are observed. The weld bond between powders and the grain boundaries inside powders are clearly observed on etched metal surface. The large powders assembled the coarse grain zone and small powders assembled the fine grain zone. The back scattered electron images (BEI) of sintered alloys obtained by SEM are shown in Figure 3 in order to reveal the detailed microstructure of sintered alloys. Even though the sintered alloys were not etched, the coarse and fine grain zones still could be distinguished in metal matrix, because the intermetallic phases in them showed different features. The grains in raw powders did not grow bigger after sintering according to Figure 1, Figure 2 and Figure 3, since the SPS procedure was performed at a relatively low temperature (500 °C) with a short dwell time (1 min), which were not enough to sustain the grain growth. However, the relatively continuous intermetallic phases along grain boundaries in unsintered powders were partially re-dissolved into alloy matrix after SPS sintering, since the sintering temperature was 500 °C, which overlapped with the solution treatment temperature (460–480 °C) of 7075 alloy [22,23]. Therefore, those intermetallic phases were broken up and showed bone-like features in coarse grain zone and speckle-like features in the fine grain zone. Besides, more and bigger black phases, which were rich in elements Mg and Si, were observed in coarse grain zone. The different features of intermetallic phases in coarse and fine grain zones could be attributed to the different cooling rate of varisized aluminium powders during atomization process, which was similar to quenching. Additionally, the SPS remained most of the quenching structures of powders in bulk alloys after sintering. The density of sintered alloys AL1 to AL6 was 2.81 g/cm^3^ according to the Archimedes test and not less than that of commercial 7075 alloy, proving that the micro-porosity, which was inevitable to traditional powder metallurgy, did not exist in SPS sintered aluminium alloys in this study.

**Figure 4:**
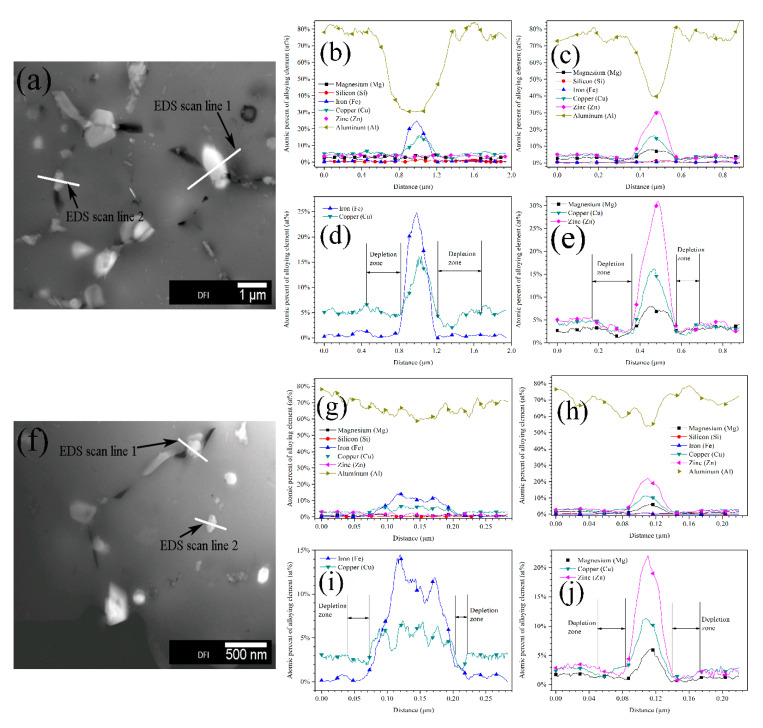
Transmission electron microscope (TEM) images of intermetallic phases in alloys matrix: (**a**) coarse grain zone, (**b**) energy dispersive X-ray spectroscopy (EDS) results of scan line 1 on coarse grain zone, (**c**) EDS results of scan line 2 on coarse grain zone, (**d**) element depletion zone near cathodic phase on coarse grain zone, (**e**) element depletion zone near anodic phase on coarse grain zone; (**f**) fine grain zone, (**g**) EDS results of scan line 1 on fine grain zone, (**h**) EDS results of scan line 2 on fine grain zone, (**i**) element depletion zone near cathodic phase on fine grain zone, and (**j**) element depletion zone near anodic phase on fine grain zone.

The TEM equipped with EDS was used to measurements in order to quantitatively analyze the diameter and microchemistry of intermetallic phases in sintered alloys. The high angle annular dark field (HAADF) images of intermetallic phases in coarse and fine grain zones as well as the EDS line scan results are shown in Figure 4. The bright-contrast phases in TEM images could be divided into two types. One type was η-phase (and its analogues) and rich in Mg, Cu, and Zn, it usually acted as anode during corrosion. The other type mainly contained Fe, Cu, and Al, and it usually acted as cathode during corrosion [10,11,18,24]. The EDS line scan results also indicate that both the anodic and cathodic phases in coarse grain zone have a bigger diameter as well as a higher alloying element content. The nucleation and growth of second phases usually result in the depletion of alloying element surrounding these phases, thus the alloying element depletion zone is always adjacent to the second phases. Even though the depletion zone is hardly observed on SEM and TEM images, it is clearly revealed by EDS linear scan, as shown in Figure 4d–j. The coarse grain zone showed a wider depletion zone with a more severe alloying element depletion when compared to the fine grain zone. While, both the anodic and cathodic phases had higher alloying element content in coarse grain zone, implying that the intermetallic phases exhibited bigger componential difference as compared to their adjacent depletion zone, which always led to more severe electrochemical heterogeneity in matrix. Table 1 lists the statistical data of intermetallic phases in coarse and fine grain zones obtained by ImageJ software. Most of the intermetallic phases distributed along grain boundaries, they exhibited more continuous feature and larger diameter in coarse grain zone, but also showed higher quantity and smaller diameter in fine grain zone.

**Figure 5:**
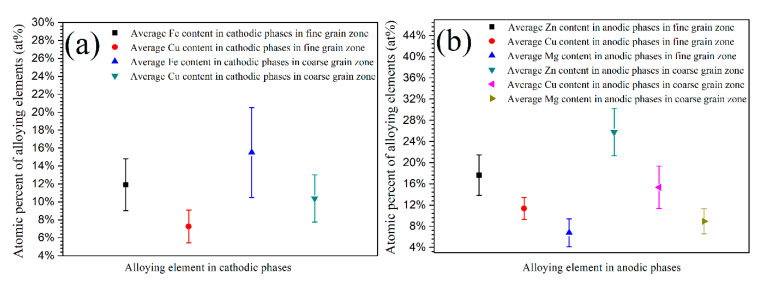
Average atomic percent of alloying elements in intermetallic phases in coarse and fine grain zones, (**a**) in cathodic phases and (**b**) in anodic phases.

Figure 5 shows the average atomic percent of alloying elements in anodic and cathodic phases obtained from numbers of EDS measurements. In the coarse grain zone, both the anodic and cathodic intermetallic phases contained more alloying elements as compared to those in fine grain zone. The microstructural and microchemical difference between coarse and fine grain zones, as shown in Figure 4 and Figure 5 and Table 1, could be attributed to the different cooling rate of large and small aluminium powders. The cooling rate of metal powders has a significant effect on the diffusion and content of alloying elements in metallic phases and depletion zone on grain boundaries. When compared to small powders, the large powders can maintain a higher temperature for a longer period due to the lower cooling rate, which are favorable for rapid diffusion of alloying elements from matrix to grain boundaries [8,9,10,11], leading to the formation of more and bigger second phases. Simultaneously, a more severe depletion of solute elements occurs inside the matrix near the grain boundaries, resulting in the formation of wide depletion zone with a low content of alloying element [10,11,21]. In addition, several small phases could grow to merge into large ones due to the precipitation and growth of second phases in coarse grains, forming more continuous and bigger phases. In contrast, fewer and smaller second phases could form during cooling in fine grain zone as a result of higher cooling rate, which suppress the diffusion of alloying elements. The effect of cooling rate on the microstructure of raw metal powders in this study is similar to that of the quench rate on aluminium alloys structure during quench process [22,23]. The microstructural and microchemical difference of intermetallic phases between large and small powders would be reserved in bulk alloys after sintering, owing to the low sintering temperature and short processing time of SPS.

The cathodic phases, which are rich in Cu and Fe in 7075 alloy, usually have a 200–600 mV higher volt potential than surrounding metal matrix. While, the anodic phases, which are rich in Zn and Mg, have a 100–300 mV lower volt potential than surrounding matrix. Additionally, the potential difference between intermetallic phases and their surrounding matrix increases with the increasing alloying elements content [25,26,27,28]. Besides, a wider alloying element depletion zone with a more severe depletion would aggravate this electrochemical heterogeneity in alloys. Therefore, the cathodic phases become more cathodic, and the anodic phases become more anodic in coarse grain zone for bulk alloys and, thus, they exhibit higher electrochemical activity and they are more sensitive to localized corrosion. Based on the above description, the intermetallic phases in coarse grain zone are more likely to lead to passive film collapse.

### 3.2. Potentiodynamic Polarization Measurement

**Figure 6:**
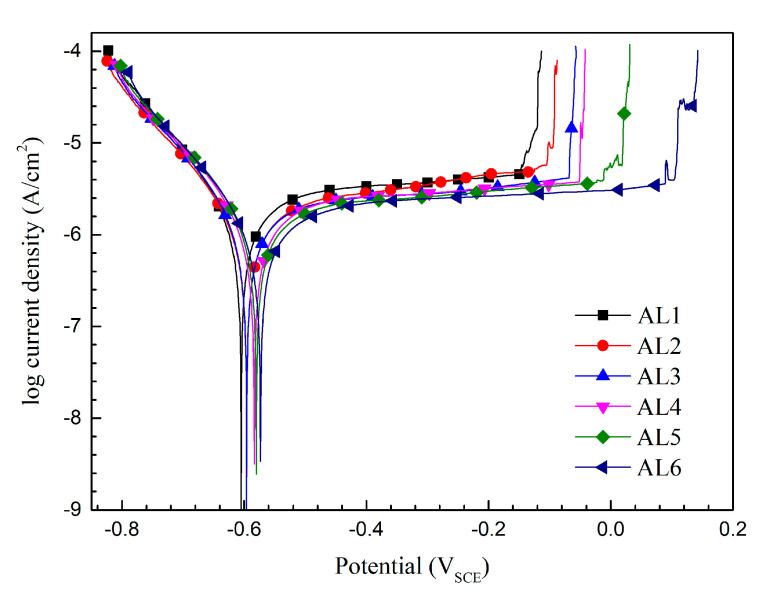
Typical potentiodynamic polarization curves of sintered 7075 alloys after 6 h immersion in 0.2 M Na_2_SO_4_ + 0.01 M NaCl solution.

Figure 6 shows the potentiodynamic polarization curves of sintered alloys after 6 h immersion in 0.2 M Na_2_SO_4_ + 0.01 M NaCl solution. All of the alloys exhibited the similar polarization behavior according to the similar shape of polarization curves. The alloys AL1–AL6 could keep passivation at corrosion potential *E*_corr_ and showed obvious passive ranges due to the tiny increasing of anodic current density with increased potential. When the applied over-potential approached dozens or a hundred of millivolts relative to the *E*_corr_, the electrode reaction current presented linear relationship with potential and, thus, approached the Tafel-type behavior. Therefore, the Tafel extrapolation was used to reveal the detailed information of polarization curves, and the relevant data are shown in Table 2. The *E*_corr_ and the pitting potential *E*_pit_ both lightly increased with the increasing content of small powders in metal matrix. While, the corrosion current density *i*_corr_ and the passive current density *i*_pass_ showed opposite changing trend as compared to those potentials, i.e., obviously decreased with small powders content, implying that fine grain zone provided a better passive capability to sintered alloys. Besides, the passive range of alloys also increased with increasing content of small powders, indicated that the passive film on fine grain zone showed a better stability. The cathodic and anodic Tafel slopes (*b*_c_ and *b*_a_) are in direct proportion to the resistance of electrode reaction [10,11]. The alloys containing more large powders (coarse grains) exhibited a smaller value of *b*_c_, implying that the bigger second phases containing higher alloying element in coarse grain zone could accelerate the cathodic reaction rate. The *b*_a_ value of alloys increased with the increasing content of small powders. A bigger *b*_a_ usually indicates a bigger dissolution resistance of passive film on metals; therefore, it could be concluded that the alloys containing more small powders have a better passive ability or a higher corrosion resistance [10,11,29], which could be also proved by the decreased *i*_corr_ and *i*_pass_ with increasing content of small powders.

The large powders had a larger volume, and thus a slower solidification and cooling rate, resulting that the intermetallic phases in them had a bigger diameter and a higher alloying elements content as compared to that in small powders, as mentioned in Section 3.1. Furthermore, the small powders had a higher grain boundary density compared to the large one. Additionally, those microstructural difference in powders were indeed retained in alloys matrix after SPS sintering. The bigger intermetallic phases usually result in a bigger cavity during localized corrosion, which will promote the stabilization of localized corrosion, such as pitting, and, thus, facilitate the fracture of passive film. While, a higher grain boundary density usually accelerates the metal atom diffusion to repair the passive film. Therefore, the sintered alloys containing more small powders exhibited a better corrosion resistance according to Figure 6 and Table 2. The volt potential difference between intermetallic phases and their surrounding matrix depends on the composition difference, and significantly increases with the increasing content of alloying elements in second phases [25,26,27,28]. Thus, coarse grain zone in sintered alloys always exhibited higher driving force to resulting in passive film fracture and localized corrosion. In contrast, the fine grain zone showed a lower electrochemical heterogeneity. Consequently, the passive properties of sintered alloys increased with the increasing content of small powders.

### 3.3. Mott-Schottky Measurement

Oxide or passive film formed on aluminium usually contains some point defects as well as a volt drop and shows semiconducting properties, which is strongly associated with the corrosion resistance of metals according to point defect model [30,31,32,33,34,35]. The defects characteristics of passive film are functions of applied potential, and do not change if the potential scan rate is fast enough (such as over 10 mV/s) [31,33,34]. Therefore, the Mott–Schottky measurement could evaluate the semiconducting properties of passive film at a fast potential scan rate. The interface capacitance of electrode system can be expressed by Equation (1) according to Mott–Schottky theory [36,37].

(1) $$\frac{1}{C^{2}}=\frac{1}{C_{H}^{2}}+\frac{2}{C_{SC}^{2}}+\frac{2}{{\varepsilon \varepsilon }_{o}eN_{A}A^{2}}\left[E-E_{fb}-\frac{kT}{e}\right]$$

where *C* is the interface capacitance of electrode system, *C*_H_ is the Helmholtz double layer capacitance, *C*_sc_ is the space charge capacitance of passive film, *e* is the electron charge (−1.6021 × 10^−19^ C), *ε*_o_ is the vacuum permittivity (8.85 × 10^−12^ F/m), *ε* is the passive film permittivity of aluminium and has a value of 10 according to literatures [38,39,40], *N*_A_ is the acceptor density (or charge carrier density in this study), *E* is the applied potential, *E*_fb_ is the flat band potential, *κ* is the Boltzmann constant (1.3807 × 10^−23^ J/K), *T* is the kelvin temperature, and *A* is the area of electrode. The Helmholtz double layer capacitance of metal has a value of 30 to 50 μF/cm^2^ and it is far bigger than the space charge capacitance of passive film of aluminium. Additionally, the high frequency (over 1 to 10 Hz) of AC amplitude also minimize the effects of *C*_H_ on *C*^−2^-potentials curves. Thus, the *C*_sc_ ≈ *C* during Mott–Schottky test, and Equation (1) can be simplified into Equation (2) for n-type semiconductor, and Equation (3) for p-type semiconductor [36,37,38,39,40].

(2) $$\frac{1}{C^{2}}=\frac{2}{{\varepsilon \varepsilon }_{o}eN_{A}A^{2}}\left[E-E_{fb}-\frac{kT}{e}\right]$$

(3) $$\frac{1}{C^{2}}=\frac{-2}{{\varepsilon \varepsilon }_{o}eN_{A}A^{2}}\left[E-E_{fb}-\frac{kT}{e}\right]$$

**Figure 7:**
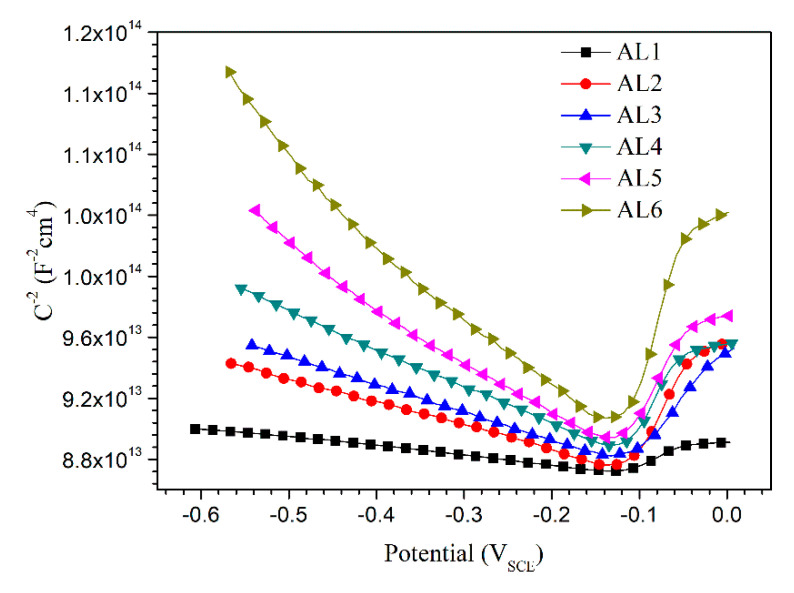
Typical Mott–Schottky test curves of sintered 7075 alloys after 6 h immersion in 0.2 M Na_2_SO_4_ + 0.01 M NaCl solution.

Figure 7 shows the Mott–Schottky plots of passive films formed on AL1to AL6. All of the plots exhibited a linear relationship between *C*^−2^ and applied potentials in the approximate potential range of −0.5 V to −0.1 V. The negative slopes of curves indicated that the passive films formed on sintered alloys showed p-type semiconductor properties in 0.2 M Na_2_SO_4_ + 0.01M NaCl solution [36,37,38,39,40]. Therefore, the charge carrier in passive film was acceptor, which was mainly represented by Al^3+^ vacancy in this study. The semiconducting type of passive film was mainly determined by chemical component of alloys, and it is not affected by the aluminium powder size. The linear relation between *C*^−2^ and potential indicates the existence of space charge layer in passive films [38,39,40], and if let the slope in Equations (2) and (3) to be *m*, then the acceptor density (or charge carrier density) *N*_A_ can be expressed by Equation (4).

(4) $$N_{A}=\frac{2}{{\varepsilon \varepsilon }_{o}em}$$

**Figure 8:**
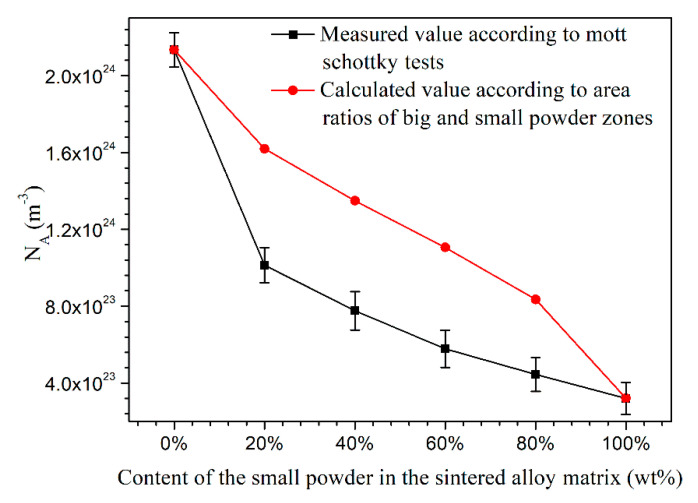
Point defects density in passive films formed on sintered 7075 alloys after 6 h immersion in 0.2 M Na_2_SO_4_ + 0.01 M NaCl solution, the standard deviation comes from five valid parallel results.

Figure 7 also shows that the absolute value of curve slope increases with the increasing content of small powders in metal matrix. The numerical data of *N*_A_ calculated according to Figure 7 and Equation (4) is shown in Figure 8 by the black line. It shows that the *N*_A_ of passive film decreases with the increasing content of small powders, indicating that the passive film that formed on fine grain zone had less point defect than that formed on coarse grain zone. A higher charge carrier density implies a highly disordered structure in passive films [36,37,38,39,40], and more point defects in passive film usually means worse corrosion resistance [41,42]. In this study, the main point defect in passive film was assigned to Al^3+^ vacancy, which has been reported to increase the conductivity of passive films and, thus, lead to a higher passive current density. This could illustrate why the alloys containing more large powders showed higher passive current density during potentiodynamic polarization. The Mott–Schottky measurements indicated that the passive film that formed on fine grain zone showed better stability and lead to a better corrosion resistance of alloys.

The small and large powders as well as the sintered alloys had identical chemical composition. It is reasonable to suggest that the difference of semiconducting properties (such as *N*_A_ value) of passive film can be attributed to the difference of grain size and intermetallic phases features. In the sintered alloys, the fine grain zone showed a far smaller grain size as compared to the coarse grain zone and, thus, contains far more grain boundaries that could accelerate the Al^3+^ cation diffusion from metal lattice to passive film. Consequently, promoted the repair of passive film on sintered alloys. In this study, the point defect in passive film was mainly Al^3+^ cation vacancy, while a faster Al^3+^ cation diffusion rate could reduce those vacancies. The same phenomena of improved passive film were also observed on refine-grained aluminium and stainless steel processed by ECAP, surface rolling, and ion sputtering [43,44,45,46]. The higher alloying element content, bigger area fraction, and diameter of intermetallic phases in coarse grain zone would increase the disordered structure, such as ion vacancy and micro-crack in passive film, and, thus, increased the point defects. Ryl et al. found that the altitude difference between second phases and their surrounding matrix was several to dozens of nanometers after polishing because of the hardness difference between them, which was determined by original diameter of second phases [47]. A large intermetallic phase usually results in a large altitude difference between second phases and their surrounding metal matrix and, thus, causes weak interface between second phases and passive film, which should be also responsible for the bigger *N*_A_ in passive film formed on coarse grain zone.

AL1 and AL6 just contained pure large and small powders, respectively. AL2 to AL5 contained both large and small powders. The area ratios of coarse grain zone to fine grain zone in alloys AL1 to AL6 were 1:0, 0.72:0.28, 0.57:0.43, 0.43:0.57, 0.28:0.72, and 0:1, respectively. If the passive film that formed on large and fine grain zones does not affect each other, the *N*_A_ should exhibit a linear relationship with the area fractions of different grain zones, and Equation (5) can be deduced.

*N*_A_ = *N*_Ab_ × *A*_b_ + *N*_As_ × *A*_s_ (5)

where *N*_Ab_ and *N*_As_ are the acceptor density of passive films on pure coarse (AL1) and pure fine (AL6) grain zones, respectively; *N*_A_ is the acceptor density of passive film formed on one electrode surface; and, *A*_b_ and *A*_s_ are the area fractions of coarse and fine grain zones on one electrode surface, respectively. The calculated *N*_A_ values of alloys is shown in Figure 8 by the red line. The measured *N*_A_ values of AL2 to AL5 were smaller than the theoretically calculated ones, which indicated that the mixture of large and small powders could generate some electrochemical interactions between each other and, thus, reduce the *N*_A_ value and improve passive properties of oxide film. The real reason of the passive film improvement caused by mixing the large and small powders is still not clear at present, because of the limitation of the current research methods.

### 3.4. Electrochemical Impedance Spectra Measurement

**Figure 9:**
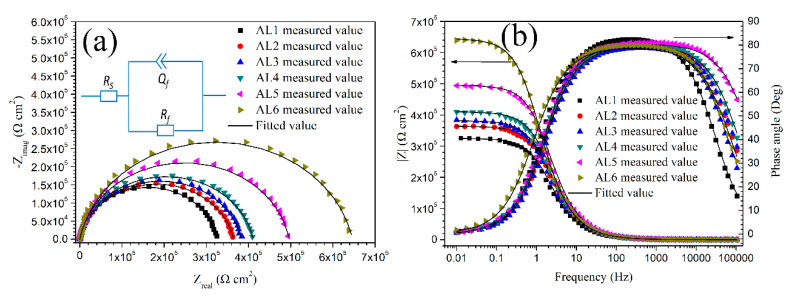
Typical electrochemical impedance spectroscopy (EIS) results of sintered 7075 alloys after 6 h immersion in 0.2 M Na_2_SO_4_ + 0.01 M NaCl solution, (**a**) Nyquist plots, (**b**) Bode plots; equivalent circuit used to fit the EIS data is shown in (**a**), *R*_s_ is solution resistance, *R*_f_ is passive film resistance, and *Q*_f_ is associated with passive film capacitance.

The EIS measurement was applied in order to evaluate the capacitance and resistance of passive film in 0.2 M Na_2_SO_4_ + 0.01 M NaCl solution. Figure 9 shows the relevant Nyquist and Bode plots. All of the sintered alloys showed a similar response during EIS tests, i.e., a depressed capacitance arc at testing frequency range, which indicated that all of the alloys formed a relatively homogenous oxide film in testing solution [11]. While the increased radius of Nyquist loop with increasing content of small powders implied that the alloys containing more small powders showed a bigger passive film impedance, which has been also proved by the |Z| value in the Bode plot. A bigger impedance usually means a better corrosion resistance of passive film. The Bode plot for each alloy showed essentially one time constant, which meant that the impedance response comes from just one interface. Two (or more) time constants would occur on bode plots if the metals encounters corrosion, one comes from passive or oxide film and the other comes from actively corroded metal interface [10,11]. The one time constant in this work mainly indicated the electrochemical response of passive film, which indicated that the passive films on all of the sintered alloys kept steady and without corrosion during EIS measurement. Therefore, the equivalent circuit, as shown in Figure 9a, was adopted to fit the EIS spectra in this work, which has been used in literatures to deduce the passive film parameters on aluminium alloys [38,47]. In the equivalent circuit, *R*_s_ is the solution resistance, *R*_f_ is the passive film resistance, and *Q*_f_ is the constant phase element, which describes the dielectric properties of passive film. The impedance of *Q*_f_ can be described by the equation *ZQ*_f_ = *Y*^−1^(*jω*)^−n^, *Y* is scaling factor, *j* is imaginary unit, and *ω* is the angular frequency. In many cases, *Q*_f_ replaced *C*_f_ (film capacitance) to provide a good fit to multiphase alloys, because it is related to the presence of different components with different capacitances [38,42,47,48]. The passive film capacitance *C*_f_ can be calculated by Equation (6)

(6) $$C_{f}=Y^{\frac{1}{n}}R^{\frac{1-n}{n}}$$

**Figure 10:**
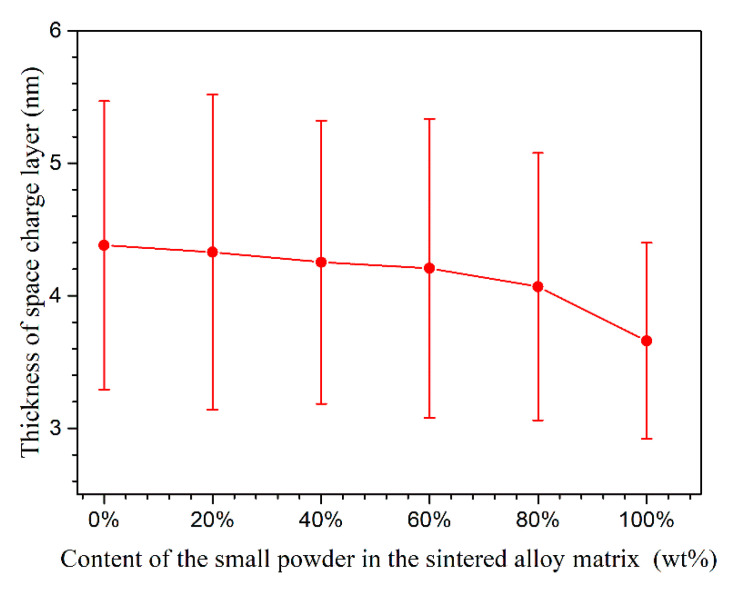
The thickness of space charge layer of passive film formed on sintered 7075 alloys; the standard deviation comes from five valid parallel results.

Table 3 lists the fitted results of EIS plots about the passive film properties. They also demonstrate that the alloys containing more small powders have a more protective passive film, since the *R*_f_ increases significantly with the increasing content of small powders. The film resistance is inversely proportional to the corrosion rate, and a bigger one usually implies good corrosion resistance of films or metals. The above results were consistent with the potentiodynamic polarization curves. The *n* values of sintered alloys were all above 0.9, which meant a good capacitive character of passive film interface [47,48]. The thickness of space charge layer *L*_ss_ can be deduced by Equation (7) [36,49].

(7) $$L_{SS}=\frac{{\varepsilon \varepsilon }_{o}A}{C_{f}}$$

where *A*, *ε*, *ε*_o_, and *C*_f_ have been mentioned above. Figure 10 shows the calculated *L*_ss_ of sintered alloys. Even though the thickness difference was not such significant, it still showed a clear changing trend. The thickness of space charge layer decreased with the increasing content of small powders. The thickness of space charge layer is proportional to the film thickness; therefore, a thicker space charge layer indicates a thicker passive film, which should provide a better protection to sintered alloys from corrosion. Besides, a thicker space charge layer should provide a longer diffusion path and, thus, slow down the carrier flow from metal surface to electrolyte. These properties would promote the corrosion resistance of passive film [30,31,32,33,34,35]. However, the polarization tests, Mott–Schottky tests, and EIS measurements all proved that the alloy containing more small powders had a more protective passive film. Therefore, the *N*_A_ value had a more dramatic impact on the corrosion resistance of passive film than the thickness of space charge layer in this study. The intermetallic phases in coarse grain zone were bigger and contained more alloying elements, and they usually resulted in more and bigger weak interfaces between them and passive film, which would facilitate the hydration of passive film during immersion [10,11,47]. This may be a possible reason for passive film on coarse grain zone having a thicker space charge layer but a worse corrosion resistance.

### 3.5. X-ray Photoelectron Spectroscopy Measurement

**Figure 11:**
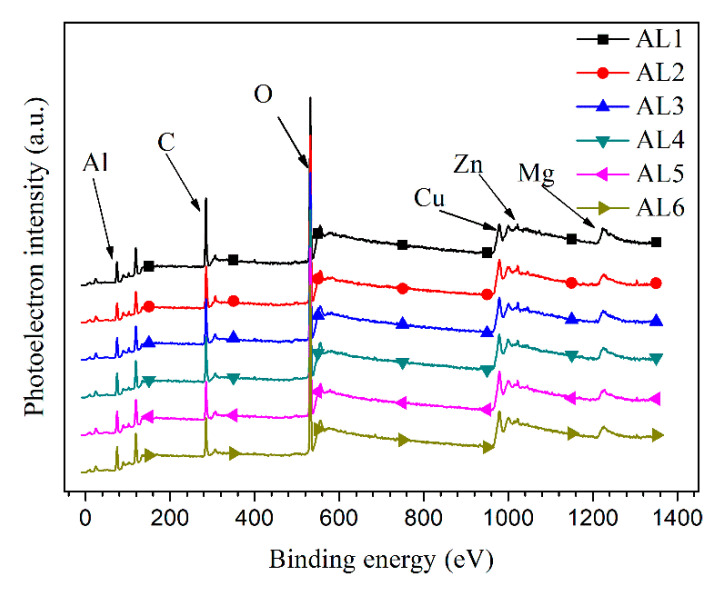
Survey X-ray photoelectron spectroscopy (XPS) spectra of the surface of sintered 7075 alloys.

**Figure 12:**
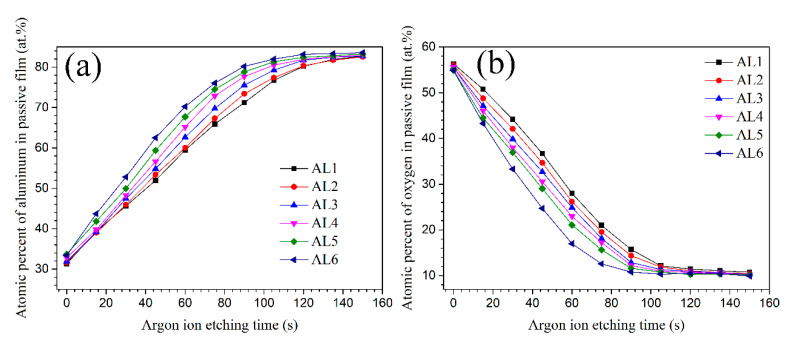
XPS depth profile of element strength of Al and O for passive film formed on sintered 7075 alloys, (**a**) Al2p, (**b**) O1s.

The XPS measurements were also performed to further characterize the passive film properties of sintered alloys, and the relevant results are shown in Figure 11. All of the sintered alloys expressed the extremely similar XPS spectra, which clearly showed the peaks of Al2p, O1s, Mg1s, Zn2p, and Cu2p and C1s [38,47,50,51]. The surface XPS spectra did not reveal the effects of powders size on the properties of passive film; therefore, the depth profiling of XPS performed by an Ar^+^ ion gun, and the etched square was 500 μm × 500 μm. Figure 12 shows the atom percent of Al (Al2p) and O(O1s) with etching time. The growth and thickening of passive film can be treated as a diffusion of oxygen atom and Al^3+^ cation with opposite diffusion direction, hence the oxide/air interface have the highest oxygen concentration and the oxide/metal interface had the highest Al concentration. Therefore, the aluminium atom percent increased, while the oxygen atom percent decreased with the etching time. According to some references, when the oxygen atom percent decreased to about 10% (or the aluminium atom percent increased to about 80%), it could be assumed that the etching have arrived to alloys substrate [28]. A longer etching time to arrive the alloys substrate means a thicker passive film. Unfortunately, the precise thickness difference of passive film for each alloys was not known, since the accurate thickness for each ion etching pass was not accurate. The AL1 and AL6 showed obvious different element distribution on thickness profile of passive film, as shown in Figure 12. The coarse grain zone formed a thicker oxide film, and the thickness of alloys passive film increased with the increasing content of large powders, which was consistent with the thickness of space charge layer for passive film. While the above conclusion disagreed with some literatures, in which they claimed that refined grains would increase the thickness of passive film [43,44,45,46]. For aluminium and its alloys, their passive film growth rate and thickness are usually determined by grain boundary density, at which the Al^3+^ cations have a faster diffusion rate and thus promote the thickening of passive film [43,44,45,46]. The fine grain zone in sintered alloys had smaller grains and higher density of grain boundary, it should have a thicker passive film. However, the XPS results in this work implied that the large electrochemical heterogeneity caused by intermetallic phases was the major driving force to passive film growth. In the other words, the intermetallic phases had a bigger effect on passive film thickening than the grain boundary density. The huge microstructural and microchemical difference between intermetallic phases and their surrounding matrix accelerated the passive film growth, however with more point defects on coarse grain zone. The alloys containing more large powders had a thicker passive film but worse corrosion resistance, which again approved that charge carrier density (*N*_A_) was the decisive factor to the corrosion resistance of passive film on sintered alloys.

For the pure aluminium, the grain boundaries usually act as atom diffusion pass and, thus, sustain the growth and repair of passive film, so that a thicker oxide film usually forms on fine-grained aluminium. However, in the aluminium alloys, the intermetallic phases usually dramatically affect the passive film growth and formation. Ryl et al. found that, in air at 50% relative humidity, the anodic phase would be firstly corroded under thin liquid film, and then the corrosion products covered on them and gradually formed a protective oxide film [47]. While the cathodic phases acted as passive film nucleation site, since oxygen reduction usually carried out on them, and the oxide film preferentially initiated as well as thickened in the immediate vicinity of them and then propagated to near area. In this study, the intermetallic phases in coarse grain zone had bigger diameter and contained more alloying element, which would increase the electrochemical heterogeneity between them and their surrounding matrix. Therefore, the electrochemical reaction, such as oxygen reduction that occurred on second phases, would be promoted and accelerated on coarse grain zone, resulting in the formation of a thicker passive film [10,11,25,26,27,28]. However, coarse grain zone still exhibited worse corrosion resistance as compared to fine grain zone, since the oxide film that formed on them contained far more point defects, which always acted as charge carrier.

### 3.6. Atomic Force Microscope Measurement

**Figure 13:**
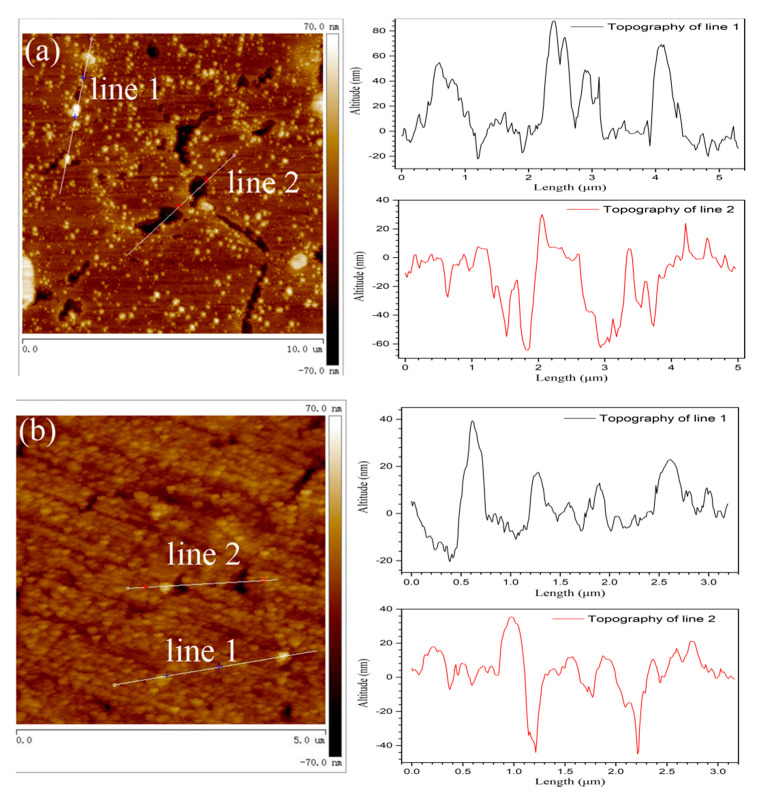
Atomic Force Microscope (AFM) images of 30 days air-exposed alloys after 1 h immersion in 3.5%wt NaCl solution, (**a**) coarse grain zone, and (**b**) fine grain zone.

The polished alloys were exposed in air for 30 days and then immersed in 3.5% NaCl solution for 1 h in order to investigate the breaking down of passive film. AFM was used to measure the morphologies of sintered alloys after immersion testing. Figure 13 shows the relevant morphologies of coarse and fine grain zones after pitting initiation. The roughness of coarse grain zone was higher than that for fine grain zone. The particles above the metal substrate were intermetallic phases, which usually had a bigger hardness when compared to metal matrix. Obviously, the intermetallic phases in coarse grain zone exhibited a higher altitude and a bigger diameter than that in fine grain zone, which agreed with the SEM and TEM results. Besides, the pits initiated around intermetallic phases were also observed in AFM images and they showed a bigger diameter and a deeper depth on coarse grain zone. The higher electrochemical activity of intermetallic phases caused by bigger diameter and more alloying element would promote the film dissolution and accelerate the pits initiation as well as propagation. As mentioned in the above sections, the intermetallic phases were the initiation site of passive film in air, yet they were also the starting site of corrosion in aerated NaCl solution. Therefore, the big intermetallic phases in coarse grain zone promoted the film thickening in air, but also accelerated the corrosion nucleation and propagation in NaCl solution with the cooperation of Cl^−^ ions.

A bigger volt potential difference caused by bigger difference of chemical composition between intermetallic phases and surrounding matrix could obviously increase the electrochemical reaction rate [10,11]. The reaction is metal dissolution for anodic phases, and it is oxygen reduction for cathodic phases. The localized solution alkalization near the cathodic phases caused by oxygen (or water) reduction could strongly dissolve the surrounding passive film. Even though the coarse grain zone had a thicker passive film, the large and high active intermetallic phases on them increased the point defect in passive film and promoted the corrosion initiation and propagation, and, thus, they showed a worse corrosion resistance than fine grain zone. The passive performance of passive film and the corrosion resistance of sintered alloys both increased with the increasing content of small powders.

In conclusion, the coarse grain zone had bigger intermetallic phases containing higher alloying element, and showed wider alloying element depletion zone with more severe element depletion compared to fine grain zone. The microstructure and microchemistry led to a bigger electrochemical heterogeneity in coarse grain zone, which resulted in the formation of a thicker passive film containing more point defects. The corrosion resistance of alloys increased with increasing content of fine grains, indicating that the passive film resistance was mainly determined by point defect density rather than the film thickness.

## 4. Conclusions

The completely densified bimodal-grain-size 7075 aluminium alloys containing varied ratios of large and small 7075 aluminium powders were prepared by SPS under the 500 °C sintering temperature and 60 MPa pressure. The large powders were 100 ± 15 μm in diameter and they constituted coarse grain zone, while the small powders were 10 ± 5 μm in diameter and constituted fine grain zone in sintered alloys. The microstructural and microchemical difference between large and small powders was remained in coarse and fine grain zones in sintered alloys due to the short cycle time of SPS.

The coarse grain zone showed bigger grain size and lower grain boundary density as compared to fine grain zone. The average diameter of intermetallic phases was 201.3 nm in coarse grain zone, while its vale was 79.8 nm in fine grain zone. The alloying element content in intermetallic phases in coarse grain zone was 33% to 48% higher than that in fine grain zone. The alloying element depletion zone adjacent to intermetallic phases in coarse grain zone showed a bigger width and more severe element depletion. The coarse grain zone in alloys exhibited bigger electrochemical heterogeneity when compared to fine grain zone.

The passive film that formed on coarse grain zone had a thicker thickness and a point defect density of 2.4 × 10^24^ m^−3^, while the film on fine grain zone had a thinner thickness and a point defect density of 4.0 × 10^23^ m^−3^. The thickness of space charge layer of passive film to sintered alloys decreased with the increasing content of fine grains. The film resistance was 3.25 × 10^5^ Ωcm^2^ on coarse grain zone, while it was 6.46 × 10^5^ Ωcm^2^ on fine grain zone. The passive potential range of sintered alloys increased from 457 mV to 678 mV, while the corrosion current density decreased from 8.59 × 10^−7^ A/cm^2^ to 6.78 × 10^−7^ A/cm^2^ as the fine grain zone increased from 0% to 100%, which implied that the corrosion resistance of alloys increased with the increasing content of fine grains. The passive film on coarse grain zone exhibited bigger corrosion cavities after pitting initiation as compared to that on fine grain zone. The passive film formed on fine grain zone showed a better corrosion resistance. The protectiveness of passive film was mainly determined by defect density rather than the thickness in this work.

## Figures and Tables

**Table 1:** Statistical data of intermetallic phases in coarse and fine grain zones in sintered alloy.

Intermetallic Phases Data	Population Density (count/μm^2^)	Area Fraction (%)	Average Phase Diameter (nm)
coarse grain zone	0.66	4.78	201.3
fine grain zone	1.23	2.35	79.8

**Table 2:** Tafel extrapolation results of potentiodynamic polarization curves of the sintered alloys, the standard deviation comes from five valid parallel results.

Alloy	*i*_corr_ (10^−7^A/cm^2^)	*b*_c_ (mV/dec)	*b*_a_ (mV/dec)	*E*_corr_ (mV_SCE_)	*E*_pit_ (mV_SCE_)	*E_pit_* − *E_corr_* (mV)
AL1	8.59 ± 0.86	122 ± 7	557 ± 23	−610 ± 2.7	−153 ± 5.1	457
AL2	8.22 ± 0.97	128 ± 6	587 ± 23	−598 ± 3.6	−124 ± 4.8	474
AL3	7.82 ± 1.06	131 ± 5	610 ± 24	−595 ± 3.8	−68 ± 6.7	527
AL4	7.57 ± 0.79	133 ± 7	645 ± 26	−589 ± 3.1	−51 ± 6.2	538
AL5	7.42 ± 0.92	134 ± 7	696 ± 21	−581 ± 2.9	20 ± 5.9	601
AL6	6.78 ± 0.89	140 ± 5	801 ± 33	−573 ± 2.3	105 ± 5.5	678

**Table 3:** The fitted results of EIS plots about the passive film formed on sintered alloys in 0.2 M Na_2_SO_4_ + 0.01 M NaCl solution, the standard deviation coms from five valid parallel results.

Alloy	*R_sol_* (Ωcm^2^)	*Y* (Scm^−2^s^n^)	*n*	*R_f_* (Ωcm^2^)	*C_f_* (Fcm^−2^)
AL1	18.58 ± 2.35	(3.11 ± 0.21) × 10^−7^	0.9202 ± 0.011	(3.25 ± 0.18) ×10^5^	(2.55 ± 0.63) × 10^−7^
AL2	21.75 ± 1.96	(3.16 ± 0.24) × 10^−7^	0.9133 ± 0.009	(3.65 ± 0.26) ×10^5^	(2.57 ± 0.70) × 10^−7^
AL3	16.31 ± 2.54	(3.24 ± 0.26) × 10^−7^	0.9083 ± 0.010	(3.83 ± 0.29) × 10^5^	(2.62 ± 0.66) × 10^−7^
AL4	19.22 ± 2.17	(3.18 ± 0.22) × 10^−7^	0.9173 ± 0.007	(4.11 ± 0.24) × 10^5^	(2.64 ± 0.71) × 10^−7^
AL5	18.78 ± 2.66	(3.22 ± 0.25) × 10^−7^	0.9175 ± 0.009	(4.97 ± 0.26) × 10^5^	(2.73 ± 0.68) × 10^−7^
AL6	17.93 ± 2.38	(3.53 ± 0.23) × 10^−7^	0.9253 ± 0.012	(6.46 ± 0.29) × 10^5^	(3.13 ± 0.63) × 10^−7^
